# After infection with *Leishmania infantum*, Golden Hamsters (*Mesocricetus auratus*) become more attractive to female sand flies (*Lutzomyia longipalpis*)

**DOI:** 10.1038/s41598-017-06313-w

**Published:** 2017-07-21

**Authors:** T. M. Nevatte, R. D. Ward, L. Sedda, J. G. C. Hamilton

**Affiliations:** 1 Institute for Science and Technology in Medicine, Guy Hilton Research Centre, Keele University, Stoke on Trent, ST4 7QB UK; 2 Centre for Applied Entomology and Parasitology, School of Life Sciences, Keele University, Staffordshire, ST5 5BG UK; 3 Centre for Health Informatics Computation and Statistics (CHICAS), Lancaster Medical School, Faculty of Health and Medicine, Lancaster University, Lancashire, LA1 4YG UK; 4 Division of Biomedical and Life Sciences, Faculty of Health and Medicine, Lancaster University, Lancashire, LA1 4YG UK

## Abstract

In Brazil, human and canine visceral leishmaniasis is caused by infection with *Leishmania infantum*, a Protist parasite transmitted by blood-feeding female *Lutzomyia longipalpis* sand flies. The objective of this study was to determine if the odour of hamsters, infected with *Le. infantum*, was more attractive than the odour of the same hamsters, before they were infected. The attractiveness of odour collected from individual hamsters (n = 13), before they were infected, was compared in a longitudinal study, with the attractiveness of the odour of the same hamster in a Y-tube olfactometer bioassay, at a late stage of infection. The odour of six of the golden hamsters was significantly more attractive to 50% of the female sand flies at the end of infection compared to before infection and the odour of four of the golden hamsters was significantly more attractive to 75% of the female sand flies at the end of infection. These results strongly indicate that hamsters infected with *Le. infantum* become significantly more attractive to a greater proportion of female sand flies as the infection progresses.

## Introduction

In South America the sand fly species complex *Lutzomyia longipalpis* (Lutz & Neiva) (Diptera: Psychodidae) is responsible for transmission of the Protist, *Leishmania infantum* (Cunha & Chagas) (Kinetoplastida: Trypanosomatidae), the causative agent of visceral leishmaniasis (VL) a disease that is fatal if left untreated^1^. The parasites are transmitted from domestic dogs, which act as the reservoir host, to humans by blood-feeding female sand flies.

Blood-feeding insects use host odours to identify and orientate towards potential host animals. These host odours are used in combination with visual, thermal and tactile host cues. The precise contribution of each of these elements to host finding depends on the insect species involved and is modulated by a combination of intrinsic and extrinsic factors^2, 3^. Within a population of potential host animals, the attractiveness of each individual host to a haematophagous insect is different and can range from very attractive to repellent^4, 5^. This differential attraction can in part be explained by the balance between the attractive and repellent chemicals that comprise their odour profile^6^.

*Lu. longipalpis* females feed on a wide range of host animals including chickens, dogs, horses, cattle and humans^7^. They are attracted by a range of host cues including host odour^7–9^, heat and CO_2_^10^. Male *Lu. longipalpis* produce a sex pheromone which although moderately attractive by itself, is synergized by the presence of host odour, to become powerfully attractive to both females and males to the vicinity of the host animal^11^.

Some parasites are able to improve their chances of transmission by manipulating the host animal, e.g. by altering its appearance or behaviour^12^. In arthropod transmitted diseases such as Malaria, the parasite has been shown to alter the behaviour of the mosquito vector by increasing frequency and persistence of probing activity^13^ and through a heightened response to host odour^14^.

Few studies have examined the potential of the parasite to alter the odour of a host animal^3^. Yet, for insect transmitted parasites, the vector response to host odour is a key determinant of feeding success and therefore disease transmission^15^. It has long been recognised that infection may change host odour, and physicians have used this observation for hundreds of years to help diagnose parasitic infection in their human patients^16^. However, it is not clear that changes in the host odour, which may be due to altered breath volatiles or epidermal microbial flora^17, 18^ lead to change in vector response to the host.

In a preliminary olfactometer study, two Golden hamsters, *Mesocricetus auratus*, infected with a Brazilian strain of *Le. infantum* (MHOM/BR/74/PP75) were found to be more attractive to female *Lu. longipalpis* sand flies than uninfected hamsters. In addition, when the volatile odours were collected from these hamsters, and tested in a Y-tube choice experiment, they were found to be significantly more attractive than odours collected from the uninfected hamsters^19^.

Although these experiments were not longitudinal and therefore an increase in attractiveness of the individual hamsters following infection was not demonstrated, they suggested that the infected hamster’s odour was altered and led to increased attractiveness of the infected animal to the vector.

A parasite manipulation theory would predict that the odour of an animal would increase in attractiveness after infection and that peak attraction would potentially coincide with peak parasite level. Therefore, the objective of this study was to compare the attractiveness of the odour of individual host animals prior to infection with *Le. infantum* with their attractiveness at a late stage of infection, when the number of transmissible amastigote infective parasites in the circulating periphery blood is likely to be at a maximum.

## Results

This study showed that the odour of hamsters infected with the Italian strain of *Le. infantum* at a late stage of infection was significantly more attractive than odour before infection (Table 1). The odour of 6 out of the 13 hamsters had become significantly attractive compared to the uninfected hamster odour for more than ½ of the sand flies (probability > 0.9) and the odour of 4 of the 13 hamsters had become significantly more attractive for more than ¾ of the sand flies (probability > 0.9) (Table 2 and Supplementary Fig. S1).

**Table 1:** The number of female sand flies responding to late stage infection odour hamsters and before infection odour in a Y-tube olfactometer, nr = no response (the number of female sand flies that did not respond after 3 mins).

hamster				number of ♀ sand flies responding to the “before infection” and “late stage infection” odours			
	sex	status	duration of inf (d)	before infection	late stage infection	*P*	nr
1	f	inf	80	2	2	1.000	76
2	f	inf	117	9	8	1.000	63
3	f	inf	117	29	32	0.798	19
4	f	inf	180	17	53	<0.001**	10
5	f	inf	130	3	48	<0.001**	29
6	f	inf	110	17	45	0.005**	18
7	f	inf	171	15	17	0.860	48
8	f	inf	143	29	25	0.683	26
9	f	inf	100	28	24	0.678	28
10	m	inf	100	8	60	<0.001**	12
11	m	inf	82	6	36	<0.001**	38
12	f	inf	153	9	50	<0.001**	21
13	m	inf	100	20	20	1.000	40
Controls							
				**number of ♀ sand flies responding to the before inoc and 99 days odour**			
				**before inoc**	**99 days**		
14	f	inoc	99	19	13	0.377	48
15	f	inoc	99	29	25	0.683	26
16	m	inoc	99	11	12	1.000	57
17	m	inoc	99	22	19	0.755	39
18	m	not-inoc	99	14	17	0.720	49
19	f	not-inoc	99	11	13	0.149	56

The numbers responding to each side of the olfactometer were compared for each hamster using a 1-proportion exact test (*P < 0.05, **P < 0.01). The Control section of the table includes female sand fly responses to sham inoculated (inoc) or not inoculated (not-inoc) hamsters. The sex of the hamster (m/f) and the length of time for infection to reach the late stage of infection (duration of inf. days) are denoted.

**Table 2:** Relative frequency is the proportion of sand flies found in the test arm (late stage infection) compared to the total number of sand flies in the test and control arm (before infection) combined.

hamster	Relative frequency	95% credible interval	Probab	Probab	Rel.Freq >0.75
		2.50%	97.50%	Rel.Freq >0.5	
1	0.5	0.16	0.86	0.5	0.1
2	0.47	0.26	0.69	0.4	<0.01
3	0.52	0.4	0.65	0.64	<0.01
4	0.75	0.65	0.85	1	0.51
5	0.93	0.85	0.98	1	0.99
6	0.72	0.61	0.82	0.99	0.29
7	0.53	0.37	0.69	0.64	<0.01
8	0.46	0.34	0.6	0.29	0
9	0.46	0.33	0.59	0.29	0
10	0.88	0.79	0.94	1	0.99
11	0.85	0.73	0.94	1	0.93
12	0.84	0.74	0.92	1	0.95
13	0.5	0.36	0.65	0.5	<0.01
**controls**					
14	0.41	0.25	0.57	0.14	0
15	0.46	0.34	0.59	0.29	0
16	0.52	0.33	0.71	0.58	<0.01
17	0.46	0.32	0.61	0.32	<0.01
18	0.55	0.38	0.71	0.7	<0.01
19	0.54	0.35	0.72	0.65	0.01

The, larger this frequency the more attractive the hamster odour is to sand flies. Credible interval is calculated on the posterior distributions (Supplementary Fig. S1) of the relative frequency of each hamster as result of accounting for uncertainty in the test parameters. The probability that the relative frequency is larger than 50 and 75% is reported in the last 2 columns.

This is a significant result, since only the infected hamsters showed attractiveness to more than ½ (6 hamsters with probability ranging from 0.99 to 1) and ¾ of sand flies (4 hamsters with probability ranging from 0.93 to 0.99); while control hamsters attractiveness to ½ of the sand flies was never significant (the credible interval was always lower than 0.5).

It could be argued that ½ of the hamsters did not attract more than ½ of infected sand flies, and that ¾ of hamsters did not attract ¾ of sand flies. However, from an epidemiological point of view, the presence of more attractive hosts in a population, at the above proportions, is a determinant for the success of disease transmission (for example the 20/80 rule^20^).

The time before the appearance of symptoms infection was not significantly associated with the proportion of attracted sand flies (P = 0.58).

The four hamsters showing the strongest attraction when infected (i.e. hamsters 5, 10, 11 and 12) were significantly different from the rest of the hamsters. The odour of these four infected hamsters showed a 30–40% larger relative frequency of attraction of female *Lu. longipalpis* than the rest of the hamsters (Supplementary Figs S2, S3, S4 and S5). The biggest difference in the dataset is between hamster number 5 and hamster number 14 (Supplementary Fig. S2, first graph on the left in the third row).

Overall the probability that infected hamsters were more attractive than control hamsters was larger than 0.999. Infected hamsters were more attractive than control hamsters, with an estimated relative frequency of 0.69 ([0.65, 0.72] credible interval) compared to 0.48 ([0.41, 0.55] credible interval) respectively.

To establish if the hamsters that were already attractive before infection improved their attractiveness we repeated the comparison on only those hamsters to which a medium-high number of sand flies were attracted before infection (i.e. ≥11 sand flies). These hamsters still improved their attractiveness, by increasing the relative frequency from 0.42 [credible interval 0.37–0.47] to 0.58 [credible interval 0.53–0.63] (probability of relative frequency larger than 0.5 is 0.999); while the control hamsters in both the “before” and “late stage infection” categories or “not inoculated” do not show any significant change in attractiveness (probability for relative frequency larger than 0.5 after 99 days is 0.311).

Within the control group of six uninfected hamsters, four of which were inoculated with uninfected spleen homogenate and 2 that were sham inoculated, there was no change in the attractiveness of their odour over time ca. 99 days (Table 2).

Analysis of the control experiments i.e. air *vs*. air, hexane *vs*. hexane and hexane *vs*. air indicated that there was no bias in the apparatus and the homogeneity test showed that data from different days could be combined for 1-proportion test analysis.

The results indicate that the change in attractiveness was due to altered volatile odour components and was not age related as there was no difference in the age of the group of hamsters that remained unattractive (mean age = 118.3 ± 11.6d) compared to the age of the group that became attractive (mean age = 125.8 ± 14.8d (T-test = *ns*).

The response of *An. gambiae* and male *Lu. longipalpis* sand flies to infected and not-infected hamster odour was variable (Table 3). Female *An. gambiae* (79% of responders) were attracted to the uninfected odour compared to infected odour (relative frequency of 94% with credible interval [0.78, 1]). However only 18% of the 160 *An. gambiae* responded in the olfactometer and the response was significant in one of the two replicates only (Binomial Test, *P* < 0.005).

**Table 3:** Response of control insects, female *Anopheles gambiae* and male *Lutzomyia longipalpis* to odour of the before and late stage infected hamsters. Responses were compared by a binomial distribution exact test.

Insect	Y-tube expt	Number of flies to:			Observed proportion	*P* value	Relative frequency	*Credible interval*	
		before infection odour	late stage infection odour	nr				2.5%	97.5%
*Anopheles gambiae* females	1	12	6	62	0.67	0.071	0.66	0.45	0.85
	2	11	0	69	1.000	0.005**	0.94	0.78	1
*Lutzomyia longipalpis* males	1	8	17	55	0.32	0.032*	0.33	0.16	0.51
	2	20	11	49	0.65	0.150	0.64	0.47	0.79

Significant (*P < 0.05, **P < 0.01) attraction to late stage infected hamsters was found in one of the two runs but not in the other for each control insect. A Bayesian test of proportion shows that only *A. gambiae* in the second run has a frequency significantly larger than 0.5.

Overall male *Lu. longipalpis* did not prefer either uninfected or infected hamster odour (total to uninfected = 17.5%; total to infected = 17.5%). Only 35% of the male *Lu. longipalpis* responded in these experiments.

## Discussion

These results show, for the first time, that hamsters became more attractive over time, because of infection with *Le. infantum*. In a group of 13 hamsters a significant proportion became attractive after infection compared to the proportion that were attractive before infection. These results can be considered to be robust since we are accounting for uncertainty in the model parameters (see methods) of the statistical test.

A pilot scale study carried out by O’Shea *et al*.^19^, showed that hamsters infected with a Brazilian strain of *Le. infantum* were more attractive than uninfected, sex and age-matched hamsters. Their study was carried out both with live animals and odour extracts made from the headspace volatiles of the infected and uninfected hamsters, compared in a dual-choice wind-tunnel and Y-tube olfactometer respectively. In the whole animal study, a significant female sand fly response to infected hamsters was observed. However, in that study only two hamsters were used for each treatment group (infected and uninfected) and there was a consequent possibility that the difference in attractiveness observed was due to variation in the attractiveness of the individual hamsters^4, 21^, i.e. the uninfected hamsters selected for the study were already less attractive than the infected hamsters and thus the difference in observed sand fly attraction was because of this variation and not related to infection status. In O’Shea’s study the whole animal wind-tunnel studies were carried out with groups of 15–20 female sand flies (replicated 12 times). This experimental design could allow the potential interaction between the female sand flies in each replicate and also as the numbers of sand flies landing on a target area around the odour source was measured, the data could be skewed by a small number of highly active sand flies thus potentially confounding the results.

The work reported in this study overcomes the limitations of the previous work as we used a larger sample size of hamsters (n = 13) and the longitudinal study design allowed us to track the change in attractiveness of individual hamsters from before infection to late stage infection, as determined by observation of the hamster. In addition, the use of a Y-tube olfactometer allowed us to treat each individual sand fly as a replicate and thus the outcome based on the observation of a very large number of replicates is more robust than the previous study.

It could be suggested that the change in attractiveness observed in this study was related to aging of the hamsters. However, this is a less likely explanation for the observed change as the group of 6 control hamsters remained unattractive throughout the experiment plus there was no significant difference in the average age of the group of infected hamsters that were attractive compared to the average age of the infected hamsters that were unattractive. Taken together these data suggest that aging by itself is an unimportant determinant of changing attractiveness. A larger group of control hamsters would have removed any possible ambiguity.

Although a comparison between male and female hamsters is not feasible due to the small number of male hamsters sampled, this work also seems to suggest an increase in attraction in both sexes. This will need to be confirmed by additional studies which may test if enhanced attraction is also an effect of the oestrous cycle of the female hamsters which had only been used during previous experiments.

Female *An. gambiae* mosquitoes were also tested in the Y-tube olfactometer with the same entrained odour samples as the male sand flies. The two separate parts of the bioassay could not be combined because the ratios of the two runs were not homogeneous. However, female *An*. *gambiae* were attracted to the before infection odour in both parts of the experiment, suggesting that *An. gambiae* were more attracted to uninfected Golden hamsters. The response of male *Lu. longipalpis* was mixed, males were not attracted to the uninfected hamster odour on either day of the bioassay. This would not be predicted from observations of sand fly behaviour in the field, where male sand flies are normally attracted to host animals before females where they establish leks and produce sex pheromone. This combination of host odour and sex pheromone is very attractive to female *Lu. longipalpis* sand flies. This raises some intriguing possibilities. In an environment with an abundance of potential host animals *Lu. longipalpis* distribution is heterogenous, it is unclear what drives this heterogeneity and these results may suggest that male sand fly choice of host odour is a key driver of the uneven distribution as they may be responding to different odour components or different concentrations than the females.

In this study not all the infected hamsters became attractive and this may be because of genetic variability between the animals. The mechanism of metabolic translation from genes to odour is currently unknown but likely to be complex^22^. It is clear from this study that four out of 13 hamsters were very attractive and this may support a “super spreader” hypothesis as they were responsible for attracting more than ¾ of the sand flies (probability > 0.9). In addition, the overall experimental results show a clear difference in attractiveness between “late stage infection” and “before-infection” hamsters, even for those hamsters with relatively medium-high attractiveness before infections. However, individual differences in attractiveness are evident and may be due to intrinsic individual or genetic characteristics of the hamsters or it may be related to the parasite load^23^. This was not measured in the current study but infectiousness to the sand fly vector has been associated with high parasite numbers in dogs, and skin parasite loads was the best predictor of being infectious^23^. This study was designed to determine the hamster attractiveness near the end of the infection cycle however, in the future it would be interesting to correlate the change in infection load and skin parasite numbers with changes in attractiveness through the full infection cycle.

In Brazil, *Le. infantum* is a zoonosis, and the parasite is transmitted primarily among domestic dogs (*Canis lupus familiaris*) by the sand fly vector, *Lu. longipalpis*. Although *Lu. longipalpis* feeds on a variety of mammalian and avian host animals only canids are considered to play a significant role in the epidemiology of the disease and in some parts of Brazil it has been reported that up to 50% of dogs have been exposed to *Le. infantum* by the time they are 3 years old. It has now been established that dogs infected with *Le. infantum*, have a different odour profile to uninfected dogs^24^. It would therefore be interesting to determine the effect of *Le. infantum* infection on the attractiveness of canid odour to *Lu. longipalpis*.

A potential relationship between skin parasite loads and an increasingly attractive odour is also an intriguing possibility, offering a potential opportunity to rapidly identify those highly infectious individuals that may be responsible for the majority of transmission^23, 25^. Furthermore, these results suggest that infection may also add to host selection criteria. Many models have been produced to predict vectorial capacity and disease transmission but none attempt to take into account the variation in host attraction levels^26^. This work along with the other evidence highlights the necessity for these models to be reviewed and to consider the inclusion of factors such as the level of infection within the population^26, 27^.

In conclusion, our results indicate that infection with *Le. infantum* enhances the attraction of hamster host odour to the sand fly vector of this parasite and this could lead to enhanced transmission. Some of the infected hamsters were particularly attractive to the sand flies. Such findings, if they were to be extrapolated to the general epidemiology of the disease, may have profound implications for the use of mathematical models, diagnosis and control of this infection. The implications of this manipulation, whereby infected individuals are more likely to be involved in transmission of disease, might require a revision of the basic epidemiological models^28, 29^.

## Methods

### Golden hamsters and Leishmania

An Italian strain of *Le. infantum*, (MHOM/IT/95/LEM3437) supplied by the London School of Hygiene and Tropical Medicine, was used to inoculate the hamsters used in this study. Ten female and three male, 4-week old Golden hamsters were inoculated intra-peritoneally with 0.5 ml of spleen homogenate containing amastigotes obtained from a single, previously infected hamster that displayed symptoms (cachexia and ascites) of late-stage *Leishmania* infection^30^. Controls were two female and two male, 4-week old hamsters inoculated with 0.5 ml of spleen homogenate from a single non-infected hamster and an additional female and male hamster not inoculated, but treated in the same way as the infected animals.

All work with hamsters was carried out with the approval of the Keele University School of Life Sciences PhD committee and Keele University Ethics Committee. Research involving the hamsters was carried out in accordance with the guidelines and regulations of the Animals in Science Regulation Unit (ASRU) and in accordance with the terms of a regulated licence (PPL40/2693) in compliance with the UK Home Office, Animals (Scientific Procedures) Act (ASPA) regulations.

### Sand flies

The sand flies were originally collected in Jacobina, Bahia State, Brazil (40°31′ W, 11°11′S) and kept in Barraud cages (18 × 18 × 18 cm) at 27 °C, 95% RH, with a photoperiod of 12:12 (L:D)^31^. Female sand flies were routinely blood-fed on anaesthetized Golden hamsters to maintain the colony.

### Host odour entrainment

**Figure 1:**
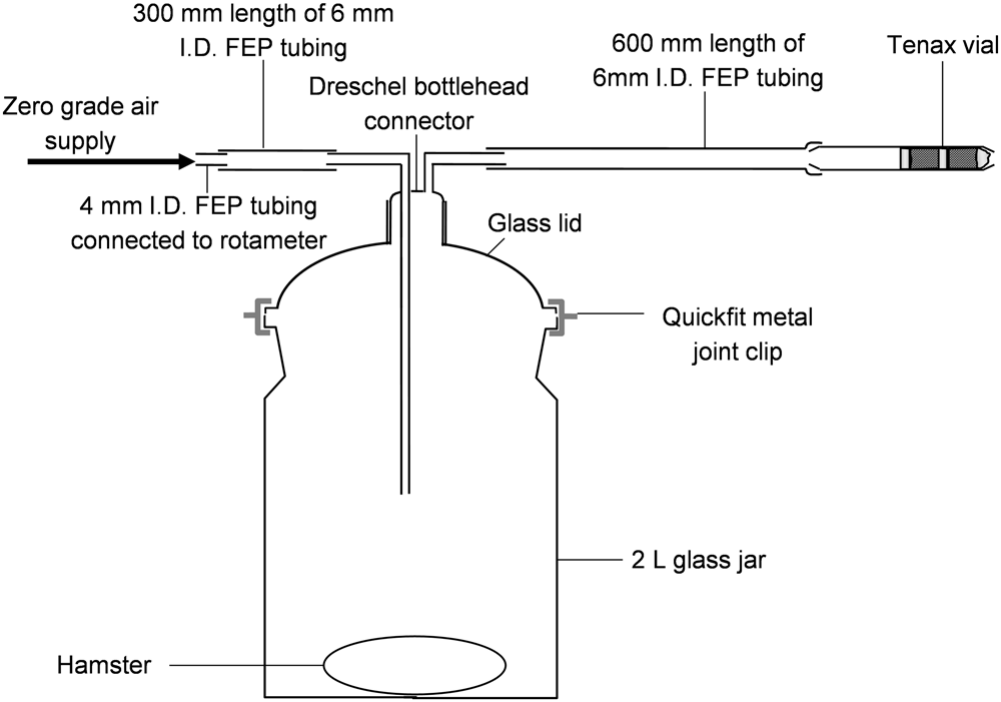
Odour entrainment apparatus. To collect the odours of *M. auratus*, the animal was placed in a 2 L glass jar; clean, zero grade air was passed over the animal and entrained odours adsorbed on Tenax TA.

Hamster odours were collected from the air passed over the animals immediately after they had been inoculated (before infection) with *Leishmania* amastigotes on 3 consecutive days for 1 hour in a volatile collection apparatus (Fig. 1). The three samples that were obtained were combined to provide the ‘before infection’ extract for each of the hamsters. The odours of the same hamsters were then collected again individually when they were nearing the end of the infection cycle (late stage infection). The late stage of infection was established by visual inspection of the hamster and by weekly monitoring of the weight of the hamster to determine cachexia (loss of weight, muscle atrophy, fatigue, weakness, and significant loss of appetite) and ascites (excess fluid build-up in the abdomen). These symptoms are indicative of late stage of infection^30^. As the infection progressed at different rates in each hamster, the odour of late stage infected hamsters was collected when they had reached the same stage of infection, ﻿(this was not dependant on chronological age of the animal) or at a pre-determined time ﻿(99 days) ﻿from inoculation (Table 1). The odour of the control animals was entrained immediately after non-infected or sham inoculation and again after 99 days.

Each animal was placed individually into a clean Quickfit culture flask (2 L) fitted with a single-port lid (Fig. 1). A Drechsel bottle head was inserted into the port and cleaned zero-grade air (7 ml s^−1^) was passed through the flask. The components of the apparatus were connected with fluorinated ethylene propylene (FEP) tubing that had been cleaned by rinsing internally before and after each entrainment with hexane (Pesticide Grade). All glassware was cleaned by first washing thoroughly with a 10% Teepol solution, rinsed with distilled water and then acetone and heated in an oven overnight at 200 °C. Airflow was periodically confirmed using a bubble-meter. The effluent air containing the hamster odour passed through an ORBO 403 filter (Tenax TA (60/80)) and after one hour the airflow was measured again and the tube disconnected and the adsorbed chemicals were eluted in hexane (1.5 ml). Control entrainments without the hamster (i.e. apparatus only) were also carried out. The volume of the entrained odour samples was reduced to 100 µl under air, and the samples stored in heat-sealed glass Pasteur pipette vials at −20 °C prior to bioassays.

### Bioassays

Six-day old, mated, sugar-starved, female sand flies were used in the Y-tube bioassays unless otherwise stated. To provide a batch of 40 female sand flies for use in the bioassays, 50 female and 20 male sand flies were collected 1-day post emergence and held together in a Barraud cage within a plastic bag (to maintain humidity), for 6 days without access to sugar. One hour before the experiment started, the sand fly holding cages were moved into the bioassay room (70 ± 5% rh; 24 ± 2 °C; fluorescent lighting), removed from the plastic bag and the sand flies allowed to acclimatise to the bioassay room conditions.

**Figure 2:**
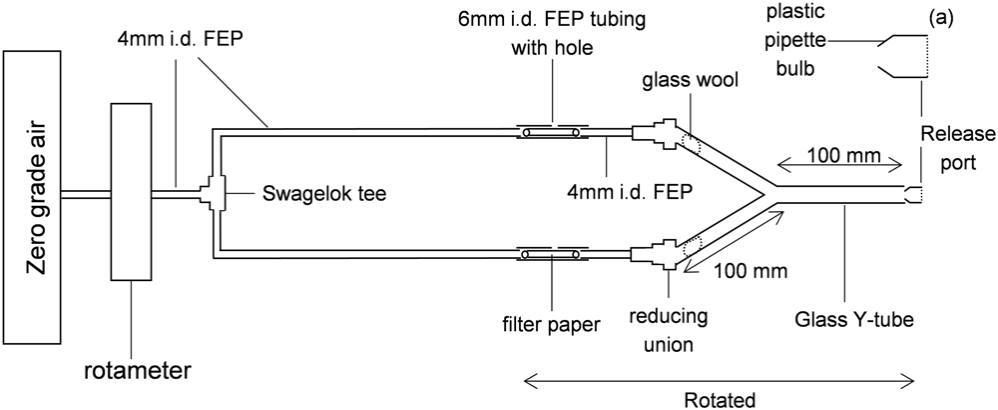
Y-tube olfactometer bioassay apparatus. A sand fly was introduced into the stem of the olfactometer using the release port shown in detail in (2a), and given time to respond to odours which had been introduced through the pierced hole onto the filter paper. Airflow was adjusted to 5 ml sec^−1^. After every ten replicates within a bioassay the Y-tube was rotated 180° around the horizontal.

A Y-tube olfactometer was used to test the attraction of individual female sand flies to the entrained odour samples^32^. The Y-tube olfactometer was formed from three lengths of glass tubing (10 mm id, ½″ od); it had two 10 cm long arms joined together at an angle of 65° with a 10 cm long stem centrally positioned between the 2 arms in the same plane to form a Y-shape (Fig. 2).

Zero-grade air was passed through a rotameter (airflow 5 ml s^−1^), and a hydrocarbon filter via FEP tubing (¼″ od). The tubing was divided into two with a brass Swagelok T-union. A 150 mm length of FEP tubing (¼″ od) was connected into each side of the T-union and these were pushed into one end of a 30mm long (¼″ id) section of FEP tubing. Each of these short sections had a hole pierced through the wall and a rolled-up, 20 mm diameter Grade 1 filter paper was inserted into it. The other end of each of the short sections of tubing was connected to a longer section of FEP tubing (40 cm long, ¼″ od) and these were connected to the arms of the Y-tube olfactometer with a brass reducing union (½″ to ¼″). Glass wool inserted into the Swagelok connector end of each Y-tube arm prevented flies from escaping. All tubing joints and connections were sealed with PTFE tape. All tubing and glassware was cleaned using the method described for odour entrainment.

To carry out the behavioural experiments the olfactometer was placed horizontally on a solid, vibration-dampened bench. Before each replicate, late stage infection extract (1 µl) was introduced into one arm of the Y-tube apparatus via the hole in the 30 mm long section of FEP tubing; the hole was then sealed with PTFE^®^ tape and before infection extract (1 µl) was introduced into the other arm. A sand fly was then removed from the holding cage and carefully transferred (Fig. 2a) into the open end of the Y-tube stem.

As soon as the sand fly had entered the Y-tube a timer was started and its final location within the Y-tube olfactometer at the end of a 3 min observation period was recorded; either in the test or control arm or, if it remained in the stem, a “no choice” was recorded.

After every 10 replicates, the rolled-up filter paper was removed from the apparatus and replaced and the whole apparatus, from the sections containing the rolled-up filter paper tubing through to and including the Y-tube, was rotated (horizontally) through 180°, so that left and right sides were exchanged. This was done to eliminate any room positional bias. Eighty female sand flies (replicates) were used for each complete experiment.

For the control experiments, one arm of the Y-tube olfactometer was used for odours entrained immediately after the sham inoculation and the other arm was used for odours entrained 99 days after the sham inoculation. Similarly, for non-inoculated hamsters, odours were collected when the hamster was 4 weeks old and then again at 99 days old.

The response of 80 female sand flies to the before infection and late stage infection entrainment samples obtained from each hamster was determined. As the experiments were conducted over 2 days (40 female replicates on each day) the results of each day’s bioassay were tested using a homogeneity test to determine if they could be combined for further analysis.

### Control olfactometer bioassays

A series of control experiments i.e. air *vs*. air, hexane *vs*. hexane and hexane *vs*. air were also carried out to check for any bias in the apparatus.

To determine if responses to entrained odour samples were specific to female sand flies alone, the Y-tube olfactometer bioassays were also carried out with female, *Anopheles gambiae* (5 day-old, un-mated, not blood-fed) and male *Lu. longipalpis* sand flies. Odour sample used for these control experiments was from hamster 12 that had previously shown enhanced attraction to female sand flies in the Y-tube bioassay. The response of 160 individual female mosquitoes or male sand flies was determined as previously described for female *Lu. longipalpis*.

### Statistical analysis

The data were analysed using both classical statistical methods; chi-squared, 1-proportion and 2-proportions tests and a Bayesian test of proportions in order to obtain probabilistic results and credible intervals from the comparison of the before infection with late stage infection results^33, 34^. Chi-square tests with exact *P* values were conducted on the individual olfactometer experiments repeated on different days to test for homogeneity before they were combined and subsequently analysed using a 1-proportion exact test to determine if there was a difference in response to the two odour sources^11^. Comparison of the change in proportion of the group of mice that were attractive before and after infection used a Fisher’s exact test of 2-proportions, the null hypothesis was that the proportion that were attractive before infection was the same as the proportion that would be attractive at late stage infection. Statistical analyses were carried out using either SPSS v14 or Minitab v17.

The exact test for proportions is a conservative statistical test as it does not take account of exogenous variability in the female sand fly frequency of response and provides only a *P*-value. Therefore, a Bayesian test of proportions was also employed. This estimated the relative frequency of sand flies in the test arm (infected odour) (*θ*) for the 13 infected and 6 control hamsters. The Bayesian model assumed that sand fly movement to one side or the other of the Y-tube is binomially distributed with parameters (*θ*, n) where *θ* itself is Beta distributed with parameters (1, 1), which is equivalent to a uniform distribution between 0 and 1 (non-informative prior). The posterior distribution is a Beta distribution with parameters *θ* + 1 and n − *θ* + 1 where n is the number of trials (i.e. the sum of the number of sand flies responding to the control and test side of the olfactometer). By employing this method, we were able to provide: the estimated relative frequency of sand flies going to the arm of the Y-tube olfactometer with late stage infection odour and its credible interval; the probability that more than 50% and 75% of sand flies moved to the late stage infection odour arm; the differences in sand fly attraction between hamsters; and the probability that infected hamsters were more attractive to sand flies than control hamsters. The Bayesian statistical analysis was carried out with the BayesianFirstAid package^35^ within the R statistical software program (R Development Core Team. 2016).

## Electronic supplementary material

Supplementary Information
