# Supplementary material for: After infection with Leishmania infantum, Golden Hamsters (Mesocricetus auratus) become more attractive to female sand flies (Lutzomyia longipalpis)
Source: Sci Rep. 2017 Jul 21;7:6104. doi: 10.1038/s41598-017-06313-w (PMC5522394; doi:10.1038/s41598-017-06313-w)
Supplement: Supplementary file 1 — Supplementary Information [file 41598_2017_6313_MOESM1_ESM.pdf]

Supplementary Information

After infection with *Leishmania infantum*, Golden Hamsters (*Mesocricetus auratus*) become more attractive to female sand flies (*Lutzomyia longipalpis*).

T.M. Nevatte, R.D. Ward, L. Sedda, and J.G.C. Hamilton

Supplementary Fig. S1

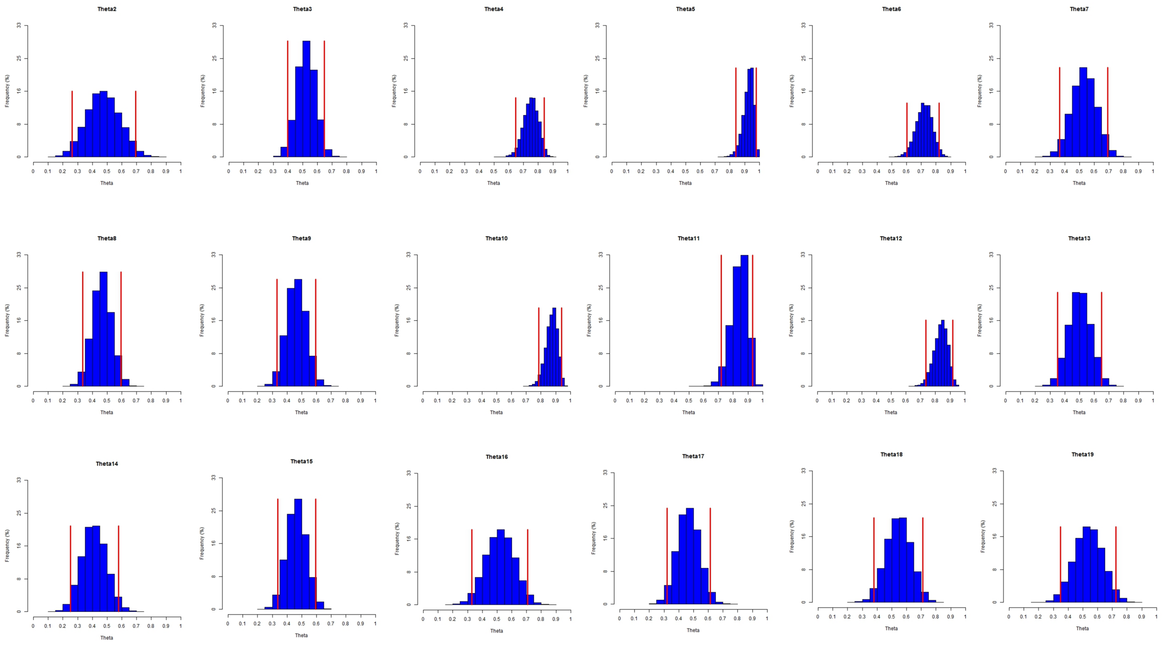

Supplementary Fig. S1: Posterior probabilities for relative frequency of sand flies found in the test arm (theta) for each individual hamster. Red lines identify the limits of the 95% credible interval.

Supplementary Fig. S2

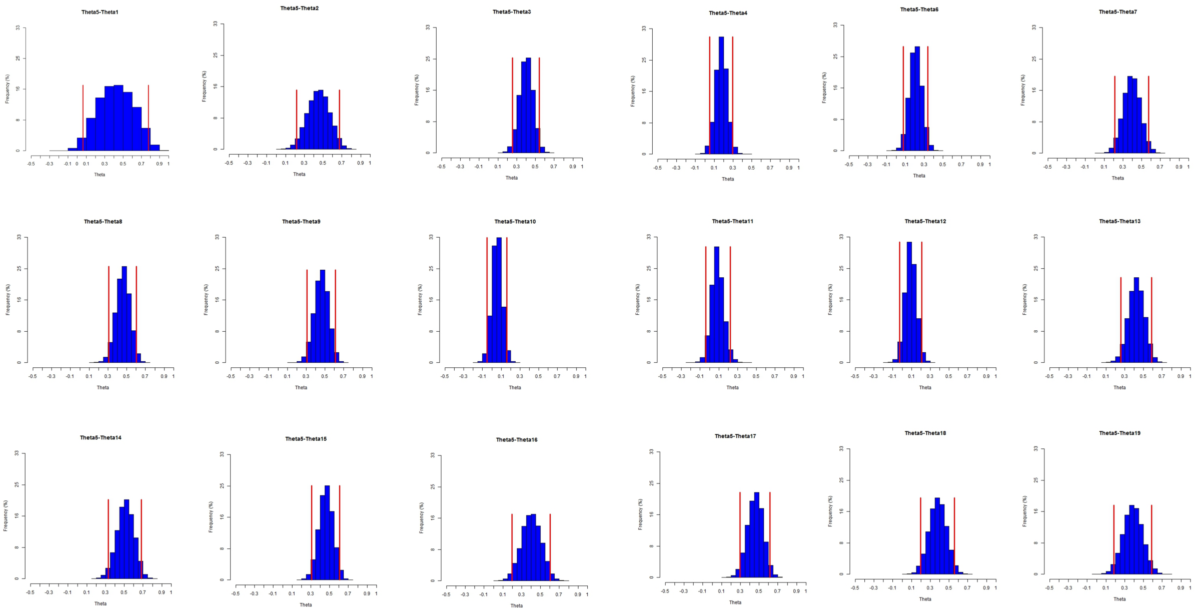

Supplementary Fig. S2: Differences in posterior probabilities for relative frequency of sand flies found in the test arm (theta) between hamster 5 and the rest 18 hamsters. Red lines identify the limits of the 95% credible interval.

Supplementary Fig. S3

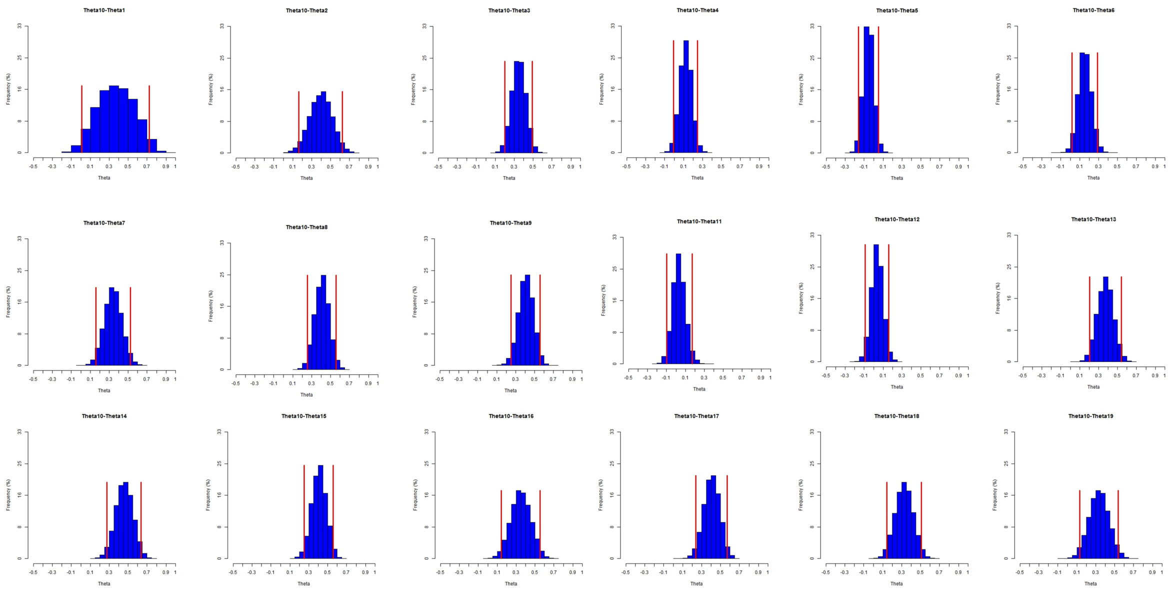

Supplementary Fig. S3: Differences in posterior probabilities for relative frequency of sand flies found in the test arm (theta) between hamster 10 and the rest 18 hamsters. Red lines identify the limits of the 95% credible interval.

Supplementary Fig. S4

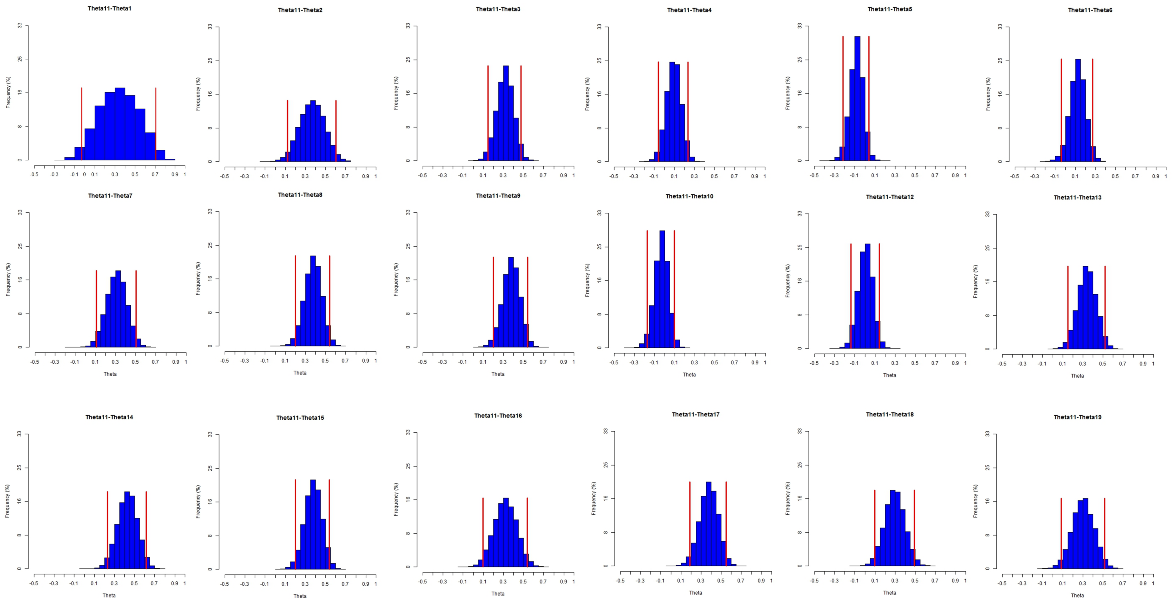

Supplementary Fig. S4: Differences in posterior probabilities for relative frequency of sand flies found in the test arm (theta) between hamster 11 and the rest 18 hamsters. Red lines identify the limits of the 95% credible interval.

Supplementary Fig. S5

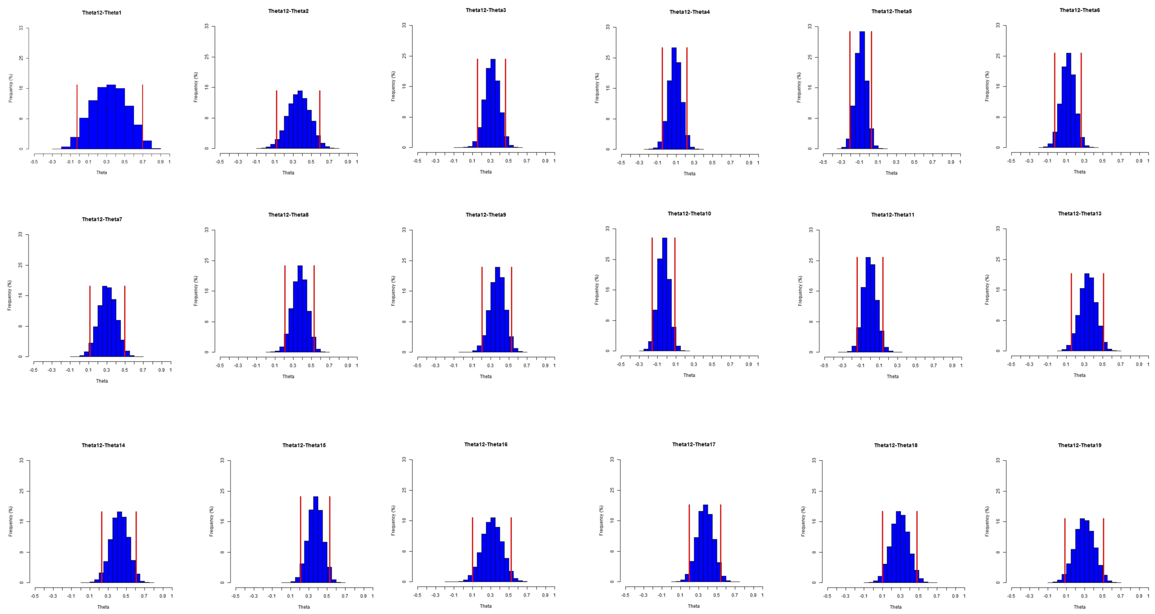

Supplementary Fig. S5: Differences in posterior probabilities for relative frequency of sand flies found in the test arm (theta) between hamster 12 and the rest 18 hamsters. Red lines identify the limits of the 95% credible interval.
